# Association of lipocalin-type prostaglandin D synthase with disproportionately enlarged subarachnoid-space in idiopathic normal pressure hydrocephalus

**DOI:** 10.1186/2045-8118-11-9

**Published:** 2014-04-15

**Authors:** Namiko Nishida, Nanae Nagata, Hiroki Toda, Naoto Jingami, Kengo Uemura, Akihiko Ozaki, Mitsuhito Mase, Yoshihiro Urade, Sadayuki Matsumoto, Koichi Iwasaki, Masatsune Ishikawa

**Affiliations:** 1 Department of Neurosurgery, Tazuke Kofukai Foundation, Medical Research Institute and Kitano Hospital, 2-4-20 Ohgimachi, Kita-ku, Osaka 530-8480, Japan; 2 Department of Neurology, Tazuke Kofukai Foundation, Medical Research Institute, Kitano Hospital, Osaka 530-8480, Japan; 3 Department of Molecular Behavioral Biology, Osaka Bioscience Institute, Osaka 565-0874, Japan; 4 International Institute for Integrative Sleep Medicine (WPI-IIIS), University of Tsukuba, 1-1-1 Tennodai, Tsukuba, Ibaraki 305-8577, Japan; 5 Department of Neurology, Kyoto University Graduate School of Medicine, Kyoto 606-8507, Japan; 6 Ishiki Hospital, Kagoshima 890-0005, Japan; 7 Department of Neurology, Saiseikai Nakatsu Hospital, Osaka 530-0012, Japan; 8 Department of Neurosurgery, Nagoya City University Graduate School of Medical Sciences, Nagoya 467-8602, Japan; 9 Department of neurosurgery and normal pressure hydrocephalus center, Rakuwakai Otowa hospital, Kyoto 607-8062, Japan

**Keywords:** Idiopathic normal pressure hydrocephalus, Frontal lobe dysfunction, CSF biomarker, L-PGDS, T-tau, White matter damage, DESH

## Abstract

**Background:**

Idiopathic normal pressure hydrocephalus (iNPH) is a treatable cause of dementia, gait disturbance, and urinary incontinence in elderly patients with ventriculomegaly. Its unique morphological feature, called disproportionately enlarged subarachnoid-space hydrocephalus (DESH), may also be a diagnostic feature. Lipocalin-type prostaglandin D synthase (L-PGDS) is a major cerebrospinal fluid (CSF) protein produced by arachnoid cells, and its concentration in the CSF is reportedly decreased in iNPH. L-PGDS acts as a prostaglandin D2-producing enzyme and behaves as a chaperone to prevent the neurotoxic aggregation of amyloid beta (Aβ) implicated in Alzheimer’s disease, a major comorbidity of iNPH. The aim of this study was to confirm the L-PGDS decrease in DESH-type iNPH and to clarify its relationship with clinico-radiological features or other CSF biomarkers.

**Methods:**

We evaluated 22 patients (age: 76.4 ± 4.4 y; males: 10, females: 12) referred for ventriculomegaly without CSF pathway obstruction, and conducted a CSF tap test to determine the surgical indication. CSF concentrations of L-PGDS, Aβ42, Aβ40, and total tau (t-tau) protein were determined using enzyme-linked immunosorbent assays. Clinical symptoms were evaluated by the iNPH grading scale, mini-mental state examination, frontal assessment battery (FAB), and timed up and go test. The extent of DESH was approximated by the callosal angle, and the severity of parenchymal damage was evaluated by the age-related white matter change (ARWMC) score.

**Results:**

L-PGDS and t-tau levels in CSF were significantly decreased in DESH patients compared to non-DESH patients (*p* = 0.013 and *p* = 0.003, respectively). L-PGDS and t-tau showed a significant positive correlation (Spearman r = 0.753, *p* < 0.001). Among the clinico-radiological profiles, L-PGDS levels correlated positively with age (Spearman r = 0.602, *p* = 0.004), callosal angle (Spearman r = 0.592, *p* = 0.004), and ARWMC scores (Spearman r = 0.652, *p* = 0.001), but were negatively correlated with FAB scores (Spearman r = 0.641, *p* = 0.004).

**Conclusions:**

Our data support the diagnostic value of L-PGDS as a CSF biomarker for iNPH and suggest a possible interaction between L-PGDS and tau protein. In addition, L-PGDS might work as a surrogate marker for DESH features, white matter damage, and frontal lobe dysfunction.

## Background

Normal pressure hydrocephalus (NPH) is a cause of treatable dementia, gait disturbance, and urinary incontinence in elderly patients with ventriculomegaly. However, the diagnostic strategy is fairly anecdotal, and ultimately dependent on successful cerebrospinal fluid (CSF) shunt surgery, particularly for idiopathic NPH (iNPH) [1,2]. Patients with several differential diagnoses such as vascular dementia, Alzheimer’s disease (AD), and other neurodegenerative disorders with lower body Parkinsonism all have the above-mentioned symptoms.

Although the clinical features may be fairly nonspecific, we must consider a CSF tap test when encountering elderly patients with dilated cerebral ventricles [3]. Analysis of CSF is sometimes helpful for estimating the underlying intracranial processes [4]. In addition to the ventriculomegaly and CSF profiles, a narrow callosal angle [5] and characteristic patterns of uneven CSF distribution within the subarachnoid space, defined as disproportionately enlarged subarachnoid-space hydrocephalus (DESH) [6], are helpful for diagnosis, and have been proposed as potential iNPH-related features.

In this study, we analyzed the clinical, radiological, and CSF profiles of 22 consecutive patients who were referred to our institute for possible iNPH. We found significant differences in the arachnoidopathic marker lipocalin-type prostaglandin D synthase (L-PGDS) between DESH and non-DESH patients. Moreover, we found that this marker was correlated with the cognitive profiles, neurodegenerative CSF markers, white matter damage scores, and tight high convexity.

## Methods

### Patients

Twenty-two patients (mean age 76.4 ± 4.4 y; 10 males, 12 females) diagnosed with possible iNPH according to Japanese guidelines [1,2], were enrolled in this study. All patients or their caregivers consented to CSF protein analysis following a tap test. This research was approved by the institutional ethics committee of Kitano Hospital.

Clinical evaluations of gait, cognition, and incontinence were performed before and 24 h after the CSF tap test, using the timed up and go test (TUG) [7], iNPH grading scale (iNPHGS) [8], mini-mental state examination (MMSE) [9], and frontal assessment battery (FAB) [10]. The patients were divided into two groups according to their radiological features: the DESH group (10 patients) and non-DESH ventriculomegaly group (12 patients) [6]. Their demographic features are summarized in Table 1. None of the patients showed the typical clinical course of AD, as diagnosed by the National Institute of Neurological Disease and Communicative Disorders and the Stroke/AD and Related Disorders Association [11]; however, some patients had been prescribed acetylcholinesterase (AChE) inhibitors for their dementia. None of the patients had an obvious history of stroke events indicative of vascular dementia (VD), or showed rigidity implicating other causes of dementia with lower body Parkinsonism.

**Table 1:** Patient characteristics

	**DESH**	**non-DESH**	***p *** **value**
Gender (male/female)	4/6	6/6	
Age (years)	75.7 ± 4.4	77 ± 9.3	0.17
Tap positive	8	5	
Shunt operation	7	3	
Shunt success	6	2	
AChE inhibitor prescription	3	5	
Opening CSF pressure (cmH_2_O)	14.8 ± 1.7	14.8 ± 1.3	0.99
Evans index (%)	34.5 ± 2.8	32.7 ± 4.3	0.27
Callosal angle (degrees)	79.9 ± 5.5	101.2 ± 4.3	0.01*
ARWMC	9.2 ± 1.6	13.3 ± 2.0	0.19
MMSE	22.5 ± 4.6	18.1 ± 6.4	0.05
FAB	11.4 ± 4.0	9.9 ± 4.0	0.27
TUG (s)	17.8 ± 0.8	29.8 ± 5.7	0.04*
TUG (step)	24.2 ± 0.6	38 ± 5.0	0.01*
iNPHGS	7.3 ± 0.9	9.1 ± 1.0	0.24
t-tau (pg/mL)	319.9 ± 31	546.2 ± 54	0.003*
Aβ42 (pM)	59.2 ± 7.5	57.2 ± 6.0	0.84
Aβ40 (pM)	1198 ± 76	1314 ± 45	0.19
L-PGDS (μg/mL)	14.4 ± 1.0	20.8 ± 2.0	0.01*

Data are numbers or the mean ± the standard deviation. iNPH: idiopathic normal pressure hydrocephalus, DESH, disproportionately enlarged subarachnoid-space hydrocephalus, AChE: acetylcholinesterase, ARWMC: age related white matter change, MMSE: mini-mental state examination, FAB: frontal assessment battery, TUG: timed up and go test, iNPHGS: iNPH grading scale, t-tau: total tau, Aβ: amyloid beta, L-PGDS: lipocalin-type prostaglandin D synthase. *: p < 0.05.

### CSF sampling and analysis

Lumbar puncture was performed in the L3–L4 or L4–L5 interspace. A 10–30-mL CSF sample was collected and gently mixed to avoid gradient effects. CSF samples with cell counts > 5/mm^3^ were excluded. All CSF samples were aliquoted and stored in polypropylene tubes at -80°C until biochemical analysis. For the CSF biomarkers, concentrations of L-PGDS, total tau (t-tau), amyloid beta (Aβ)1–42 (Aβ42), and Aβ1–40 (Aβ40) were estimated. L-PGDS levels were measured with a standardized in-house enzyme-linked immunosorbent assay (ELISA) method, as previously reported [12]. As a control group, the L-PGDS concentration in samples from 11 patients over the age of 50 was adopted from previously reported data [13]. The CSF concentration values of Aβs and t-tau were determined with standardized commercially available ELISA kits obtained from Immuno Biological Laboratories (IBL, Gunma, Japan) and Invitrogen (Invitrogen, Camarillo, CA, USA), respectively. The assay was performed according to the manufacturer’s protocol. As a control group for Aβs and t-tau, the CSF from 11 patients over the age of 60 with Parkinsonism but without radiological ventriculomegaly was used.

### Magnetic resonance imaging

A 3.0-Tesla magnetic resonance imaging (MRI) system (Achieva Quasar; Philips Medical Systems, Netherlands) was used. Three-dimensional T1-weighted fast-field echo images (repetition time [TR], 25 ms; echo time [TE], 2.2 ms; flip angle, 30°; slice thickness, 2.0 mm; intersection gap, 0.0 mm; field of view, 256 mm; matrix, 256 × 256) and T2-weighted turbo spin echo images (TR, 5477 ms; TE, 90 ms; slice thickness, 2.0 mm; intersection gap, 0.0 mm; field of view, 256 mm; matrix, 256 × 256) were obtained in sections parallel to the anteroposterior commissure plane, covering brain regions from the base of the cerebellum to the vertex. All MRI evaluations were done by the first author. Evans index was calculated as the maximal width of the frontal horns/maximal width of the inner skull [14]. For approximating DESH severity, we measured the callosal angle on coronal images perpendicular to the anteroposterior commissure plane on the posterior commissure, according to an existing protocol [5]. To evaluate the concomitant ischemic lesions in the cerebral white matter, we used the age related white matter change (ARWMC) score [15].

### Data analysis

Values are given as means and standard deviations. We compared DESH and non-DESH group parameters using the Wilcoxon signed-rank test. Comparisons among the two groups with ventriculomegaly (DESH and non-DESH; tap test positive and negative) and the control group were done by one-way analysis of variance (ANOVA) followed by post hoc Newman-Keuls multiple comparison test. The relationships among demographical, radiological, and laboratory data were evaluated by Spearman correlation tests. All statistical analyses were performed using GraphPad Prism 5.01 (GraphPad Software, Inc., La Jolla, CA, USA), and *p* < 0.05 was considered statistically significant.

## Results

**Figure 1:**
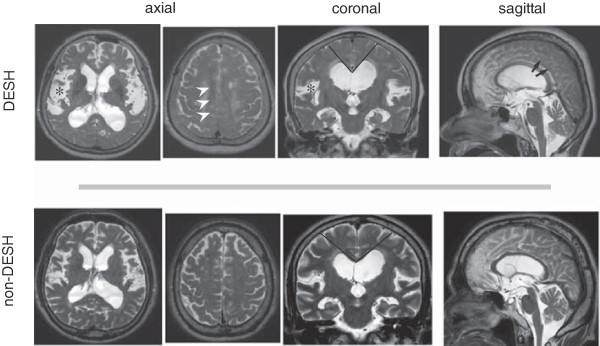
**Representative magnetic resonance images of disproportionately enlarged subarachnoid-space hydrocephalus (DESH, upper row) and non-DESH ventriculomegaly (bottom row).** Asterisks mark dilatation of the Sylvian fissure. Arrow heads, tight high convexity; Bars, callosal angles; Arrows, tight medial parietal sulci.

According to the radiological criteria, 10 of the 22 patients showed typical DESH patterns (Table 1). Representative DESH and non-DESH patterns on MR images are shown in Figure 1. Although both groups showed ventriculomegaly, uneven CSF distribution within the subarachnoid space was more prominent in DESH patients. Their demographical backgrounds, opening pressures, and the degree of ventriculomegaly as assessed by Evans index were similar (Table 1). The callosal angle, which is a quasi-quantitative representative of tight high convexity (an important factor in DESH), was significantly smaller in DESH patients compared to in non-DESH patients (DESH: 79.9 ± 5.5 degrees, non-DESH: 101.2 ± 4.3 degrees, *p* = 0.01). Eight out of 10 DESH patients showed a positive tap test response. Of these patients, 7 underwent shunt operation, and 6 responded positively to the shunt. On the contrary, only 5 out of the 12 non-DESH patients were tap test positive, with 3 undergoing surgery, and 2 being shunt responders (Table 1). Three of the DESH patients and 5 of the non-DESH patients were already prescribed AChE inhibitors for their dementia. Five of the 8 patients with AChE inhibitor prescriptions responded to the tap test and 3 of the 5 tap test responders (all with DESH features) underwent surgery with successful outcomes. ARWMC scores seemed to be worse in non-DESH patients, but this difference was not significant. The TUG test results were significantly better in the DESH patients compared to non-DESH (17.8 ± 0.8 s/24.2 ± 0.6 steps, compared to 29.8 ± 5.7 s/38 ± 5.0 steps, *p* = 0.04/0.01). Among the CSF biomarkers, t-tau and L-PGDS were significantly lower in the DESH group (t-tau: DESH: 319.9 ± 31.4, non-DESH: 546.2 ± 54.2 pg/mL, *p* = 0.003; L-PGDS: DESH: 14.4 ± 1.0, non-DESH: 20.8 ± 2.0 μg/mL, *p* = 0.01). The 8 patients with AChE inhibitor prescriptions were slightly older (with AChE inhibitors: 82.0 ± 1.6 y, without AChE inhibitors: 73.2 ± 2.0 y, *p* = 0.007) and had higher CSF tau levels (with AChE inhibitors: 546.3 ± 76 pg/mL, without AChE inhibitors: 384.5 ± 40 pg/mL, *p* = 0.05) compared to patients without AChE inhibitor prescriptions. However, their tau levels were still low compared to the institutional values for AD patients (1076.7 ± 608 pg/mL, unpublished data).

**Figure 2:**
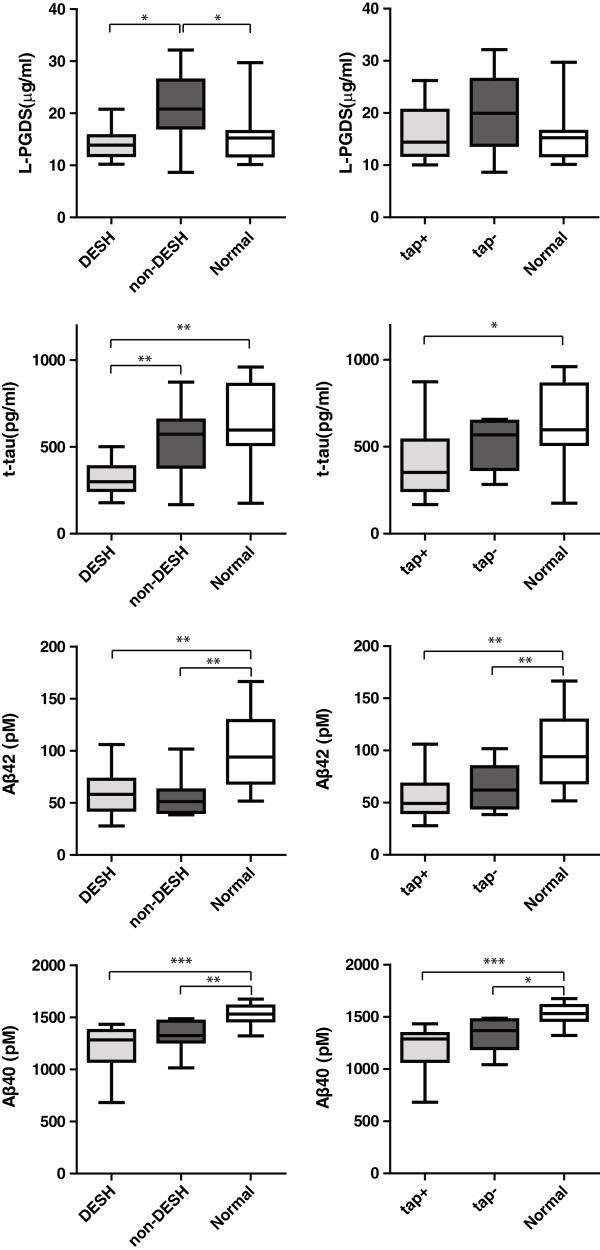
**Comparisons of CSF biomarkers among the two ventriculomegalic groups (DESH-based or tap-test-based) and control group.** Central bars: median values, box edges: range of 75 percentile, whisker edges: upper and lower limit values. Significant differences: *p < 0.05, **p < 0.01, and ***p < 0.001 by one-way analysis of variance followed by post hoc Newman-Keuls multiple comparison tests. Aβ: amyloid beta DESH: disproportionately enlarged subarachnoid-space hydrocephalus, L-PGDS: lipocalin-type prostaglandin D synthase, t-tau: total tau.

To clarify CSF biomarker differences between the two ventriculomegalic groups (dichotomized on DESH or on tap test) and non-ventriculomegalic controls, we recruited two control groups for L-PGDS and neurodegenerative markers. As shown in Figure 2, L-PGDS and t-tau discriminated DESH in ventriculomegalic patients, but did not predict the tap test results. Both t-tau and Aβ concentrations were low in the ventriculomegalic groups compared to the control group. However, Aβ concentrations did not distinguish DESH- or tap-test-based differences.

To elucidate the relationship between clinico-radiological features and CSF biomarkers further, a correlation analysis was performed. As shown in Table 2, t-tau and L-PGDS showed a significant positive correlation (Spearman r = 0.753, *p* < 0.001). Age and callosal angle correlated positively with both t-tau (age: Spearman r = 0.638, *p* < 0.002; callosal angle: r = 0.653, *p* < 0.001) and L-PGDS (age: Spearman r = 0.602, *p* < 0.004; callosal angle: r = 0.592, *p* < 0.004). L-PGDS also correlated positively with ARWMC scores (Spearman r = 0.652, *p* < 0.001) and negatively with FAB scores (Spearman r = -0.641, *p* < 0.004). ARWMC scores were negatively correlated with MMSE (Spearman r = -0.681, *p* < 0.001) and FAB (Spearman r = -0.659, *p* < 0.001) scores. Compared to other CSF biomarkers, Aβs were not correlated with clinico-radiological features at the *p* < 0.005 level.

**Table 2:** Table of correlation analyses for variables measured in study of twenty two adult patients with ventriculomegaly

	**Age**	**Evans index**	**Callosal angle**	**ARWMC**	**iNPHGS**	**TUG (s)**
Evans index	-0.049 (0.831)					
Callosal angle	0.379 (0.076)	-0.188 (0.402)				
ARWMC	0.331 (0.143)	-0.575 (0.006)*	0.357 (0.103)			
iNPHGS	0.158 (0.494)	-0.177 (0.442)	0.179 (0.426)	0.466 (0.033)*		
TUG (s)	-0.127 (0.616)	0.138 (0.584)	0.407 (0.094)	0.206 (0.411)	0.548 (0.019)*	
TUG (step)	-0.002 (0.995)	-0.001 (0.997)	0.575 (0.013)*	0.132 (0.601)	0.415 (0.087)	0.801 (<0.001)**
MMSE	-0.133 (0.564)	0.453 (0.040)*	-0.289 (0.191)	-0.681 (0.005)**	-0.638 (0.002)**	-0.290 (0.243)
FAB	-0.323 (0.153)	0.268 (0.240)	-0.192 (0.406)	-0.659 (0.001)**	-0.564 (0.008) *	-0.293 (0.238)
t-tau	0.638 (0.002)**	-0.339 (0.133)	0.653 (0.001)**	0.490 (0.024)*	0.542 (0.011)*	0.247 (0.324)
Aβ42	0.253 (0.268)	0.045 (0.845)	0.175 (0.437)	-0.252 (0.271)	-0.188 (0.415)	-0.062 (0.807)
Aβ40	0.491 (0.024)*	-0.160 (0.489)	0.560 (0.007)*	0.067 (0.774)	-0.024 (0.918)	-0.066 (0.795)
L-PGDS	0.602 (0.004)**	-0.166 (0.471)	0.592 (0.004)**	0.652 (0.001)**	0.374 (0.095)	0.144 (0.570)
	TUG (step)	MMSE	FAB	t-tau	Aβ42	Aβ40
MMSE	-0.530 (0.024)*					
FAB	-0.345 (0.168)	0.631 (0.002)**				
t-tau	0.418 (0.084)	-0.456 (0.033) *	-0.387 (0.083)			
Aβ42	0.029 (0.909)	0.416 (0.052)	0.432 (0.051)	0.104 (0.654)		
Aβ40	0.085 (0.738)	0.098 (0.567)	0.262 (0.252)	0.530 (0.014)*	0.667 (0.001)**	
L-PGDS	0.238 (0.342)	-0.460 (0.028) *	-0.641 (0.004)**	0.753 (<0.001)**	-0.058 (0.801)	0.357 (0.106)

Spearman r (*p* value). Significant correlations: *p < 0.05, **p < 0.001. ARWMC: age related white matter change, iNPHGS: iNPH grading scale, TUG: timed up and go test, MMSE: mini-mental state examination, FAB: frontal assessment battery, t-tau: total tau, Aβ: amyloid beta, L-PGDS: lipocalin-type prostaglandin D synthase.

## Discussion

In this study, we confirmed the usefulness of MRI-based (DESH-based) diagnostic schemes and recognized the lower success rate of tap tests in non-DESH ventriculomegaly. As for the CSF biomarkers, we confirmed that patients with DESH-type iNPH had significantly lower L-PGDS and t-tau levels compared to non-DESH. Moreover, we recognized the positive correlation between L-PGDS and t-tau, both of which correlated positively with the callosal angle and age of ventriculomegalic patients.

In addition to our small sample size, this study, similar to previous clinical studies on iNPH, has several further limitations that were related to the patients’ comorbidities [16]. We included 8 patients who had been prescribed AChE inhibitors in this study, yet their CSF t-tau levels were still low compared to that of full-blown AD patients. Another problem related to comorbidity was the diagnosis of VD, which was rather difficult when the patients did not have a clear history of stroke, but did have severe small vessel disease with ventriculomegaly according to MRI. In this study, we used the patients’ ARWMC scores [15] instead of several other VD diagnostic criteria [17] to represent their potential VD comorbidity. Furthermore, true iNPH patients have undergone successful shunt operations, yet many of the patients with ventriculomegaly do not reach the operation room even after a positive tap test due to comorbidities and social problems. In this regard, we arbitrarily grouped our patients using a DESH-based scheme, which was supported by the results of subsequent correlation analyses.

Several reports have suggested that the CSF biomarker t-tau plays a role in the neurodegenerative mechanisms underlying iNPH development. Tau is a microtubule-associated protein that promotes and stabilizes microtubule assembly, and is primarily located in the axons of neuronal cells [18,19]. Increases in CSF t-tau indicate the severity of neuronal damage and loss [20]. CSF t-tau increases with age and the severity of clinical symptoms in iNPH, and tends to be lower in patients with good cognitive recovery after shunt surgery [21,22]. According to our study and several previous studies on iNPH, t-tau levels in iNPH were usually around the normal range, and were significantly lower than that of AD and VD patients [23-25]. Preoperative high lumber CSF t-tau might be an ominous sign for shunt candidates.

In regards to L-PGDS, its decrease has been attributed to arachnoidopathy (i.e., loss of arachnoid cells producing L-PGDS) particularly in secondary NPH after subarachnoid hemorrhage [13,26]. In our study, decreased L-PGDS was correlated with a narrow callosal angle, which is a feature of an uneven CSF distribution in DESH-type iNPH. However, whether DESH is caused by arachnoidopathy is still under discussion. Moreover, it seemed rather paradoxical that patients with low L-PGDS levels showed high cognitive function despite the severe arachnoidopathy. Compared to previously reported control groups, L-PGDS levels in our DESH group appeared normal, while the levels in the non-DESH group were significantly higher. These findings may suggest that the non-DESH patients were simply in a later stage of hydrocephalus progression (e.g., white matter damage), and thus had less of a chance to improve with either the tap test or shunting [27].

L-PGDS is considered a dual functional protein, i.e., it acts as a prostaglandin D2-producing enzyme and as a lipophilic ligand-binding protein [28]. Its lipophilic nature allows it to function as a chaperone for preventing the formation of neurotoxic agents, such as Aβ fibrils [29]. In this regard, we initially expected there to be a relationship between CSF Aβ and L-PGDS. However, both DESH and non-DESH ventriculomegaly groups showed similar low levels of Aβs compared to the control groups without ventriculomegaly. Instead, we found a significant correlation between CSF t-tau and L-PGDS. Tau is a hydrophilic protein, yet its neurotoxic aggregation certainly occurs in AD and in several tauopathies [18]. Tau protein is greatly charged and has the capacity to interact with many partners; its interaction with L-PGDS, however, needs further investigation.

## Conclusions

In summary, we found two potential CSF biomarkers, t-tau and L-PGDS, for distinguishing DESH-type iNPH from non-DESH type. Moreover, their concomitant decrease and positive relationship could indicate an unknown mechanism underlying iNPH development. In addition, we propose L-PGDS as a surrogate marker of DESH features, white matter damage, and frontal lobe dysfunction. The same two markers did not predict tap test results.

## Abbreviations

Aβ: Amyloid beta; AChE: Acetylcholinesterase; AD: Alzheimer’s disease; ARWMC: Age related white matter change; CSF: Cerebrospinal fluid; DESH: Disproportionately enlarged subarachnoid-space hydrocephalus; ELISA: Enzyme-linked immunosorbent assay; FAB: Frontal assessment battery; iNPH: Idiopathic normal pressure hydrocephalus; iNPHGS: iNPH grading scale; L-PGDS: Lipocalin-type prostaglandin D synthase; MMSE: Mini-mental state examination; MRI: Magnetic resonance imaging; TE: Echo time; TR: Repetition time; t-tau: Total tau; TUG: Timed up and go test; VD: Vascular dementia.

## Competing interests

None of the authors have financial disclosures to declare.

## Authors’ contributions

NNi: designed the study, carried out the clinical and MRI evaluations, performed the statistical analysis, and drafted the manuscript. NNa, MM, NJ, and KU carried out the immunoassays. HT, AO, SM, and KO helped coordinate the study. MM, YU, and MI performed critical revisions of the manuscript for important intellectual content. All authors read and approved the final manuscript.
